# AHSA1 is a promising therapeutic target for cellular proliferation and proteasome inhibitor resistance in multiple myeloma

**DOI:** 10.1186/s13046-021-02220-1

**Published:** 2022-01-06

**Authors:** Chunyan Gu, Yajun Wang, Lulin Zhang, Li Qiao, Shanliang Sun, Miaomiao Shao, Xiaozhu Tang, Pinggang Ding, Chao Tang, Yuhao Cao, Yanyan Zhou, Mengjie Guo, Rongfang Wei, Nianguang Li, Yibei Xiao, Jinao Duan, Ye Yang

**Affiliations:** 1 Nanjing Hospital of Chinese Medicine affiliated to Nanjing University of Chinese Medicine, Nanjing, 210023 China; 2 School of Medicine & Holistic Integrative Medicine, Nanjing University of Chinese Medicine, Nanjing, 210023 China; 3 School of Pharmacy, Nanjing University of Chinese Medicine, Nanjing, 210023 China; 4 School of Pharmacy, China Pharmaceutical University, Nanjing, 211198 China; 5 State Administration of Traditional Chinese Medicine Key Laboratory of Chinese Medicinal Resources Recycling Utilization, Jiangsu Collaborative Innovation Center of Chinese Medicinal Resources Industrialization, Nanjing University of Chinese Medicine, Nanjing, 210023 China

**Keywords:** Multiple myeloma, AHSA1, HSP90, Bufalin, Proliferation, Drug resistance, Proteasomal inhibitor, KU-177

## Abstract

**Background:**

Currently, multiple myeloma (MM) is still an incurable plasma cell malignancy in urgent need of novel therapeutic targets and drugs.

**Methods:**

Bufalin was known as a highly toxic but effective anti-cancer compound. We used Bufalin as a probe to screen its potential targets by proteome microarray, in which AHSA1 was the unique target of Bufalin. The effects of AHSA1 on cellular proliferation and drug resistance were determined by MTT, western blot, flow cytometry, immunohistochemistry staining and xenograft model *in vivo*. The potential mechanisms of Bufalin and KU-177 in AHSA1/HSP90 were verified by co-immunoprecipitation, mass spectrometry, site mutation and microscale thermophoresis assay.

**Results:**

AHSA1 expression was increased in MM samples compared to normal controls, which was significantly associated with MM relapse and poor outcomes. Furthermore, AHSA1 promoted MM cell proliferation and proteasome inhibitor (PI) resistance *in vitro* and *in vivo*. Mechanism exploration indicated that AHSA1 acted as a co-chaperone of HSP90A to activate CDK6 and PSMD2, which were key regulators of MM proliferation and PI resistance respectively. Additionally, we identified AHSA1-K137 as the specific binding site of Bufalin on AHSA1, mutation of which decreased the interaction of AHSA1 with HSP90A and suppressed the function of AHSA1 on mediating CDK6 and PSMD2. Intriguingly, we discovered KU-177, an AHSA1 selective inhibitor, and found KU-177 targeting the same site as Bufalin. Bufalin and KU-177 treatments hampered the proliferation of flow MRD-positive cells in both primary MM and recurrent MM patient samples. Moreover, KU-177 abrogated the cellular proliferation and PI resistance induced by elevated AHSA1, and decreased the expression of CDK6 and PSMD2.

**Conclusions:**

We demonstrate that AHSA1 may serve as a promising therapeutic target for cellular proliferation and proteasome inhibitor resistance in multiple myeloma.

**Supplementary Information:**

The online version contains supplementary material available at 10.1186/s13046-021-02220-1.

## Background

Multiple myeloma (MM) is still an incurable plasma cell malignancy, though the advanced development of chemical and biological therapeutic drugs and methods, like proteasome inhibitors (PI) and Chimeric Antigen Receptor T Cell Therapy (CART) et al., has greatly improved MM overall survival (OS) [1–3]. MM patients typically responded to the initial treatments, but mostly acquired drug-resistance, failed in the treatment and died of the illness eventually. Therefore, seeking for novel therapeutic targets and developing effective drugs to overcome MM drug-resistance and relapse is of extreme importance [4–8].

Bufalin, an anticancer molecular extract from traditional Chinese medicine [9], exerts significantly experimental anticancer effects in various cancers including MM by inducing cellular apoptotic, autophagic and anti-inflammatory activities [10, 11]. However, the potential side effects and toxicities of Bufalin such as hematological, gastrointestinal, mucocutaneous and cardiovascular adverse reactions limit the clinical setting of Bufalin [12]. To avoid the side effects and toxicities of Bufalin, we designed the experiments to discover the specific target of Bufalin in MM and explore the potential anticancer effect by employing the correspondingly selective inhibitor. By using Bufalin as a probe labelled with CY5, AHSA1 was screened out as a potential target by proteome microarray combined with microarray cohorts of MM patients.

AHSA1 (Activator of HSP90 ATPase Activity 1) serving as a co-chaperone of HSP90A activates the ATPase activity of HSP90A leading to the elevation of its chaperone activity, and thereby provides a regulatory mechanism for chaperoning of client proteins [13]. HSP90A is constitutively expressed higher levels (2- to 10-fold) in cancer cells relative to their normal counterparts [14], and the interaction with its client proteins is involved in tumorigenesis through the regulation of oncoproteins including signaling proteins, receptors, and transcriptional factors [15]. For instance, mass spectrometry (MS) analysis on the pulldown-proteins with PU-H71- and GA-conjugated beads indicates that many proteins are highly enriched in key oncogenic signaling pathways, such as the PI3K–AKT–mTOR, CDK6, and MYC transcriptional activity. HSP90 can bind to these proteins and influence their conformation resulting in the activation of these pathways [16–18]. Hsp90 has been known as a cancer therapeutic target for several decades [15]. More than 20 inhibitors of Hsp90 have entered clinical trials for cancer treatment, however most of them encountered deleterious side effects and toxicities [19]. The wide spectrum inhibitory effect of HSP90 inhibitors on the client proteins is one of the primary causes for the failure of these inhibitors in the clinical trials [20]. Intriguingly, AHSA1 selectively mediates the recruitment of client proteins to Hsp90 system as an adaptor co-chaperone suggesting the therapeutic potential of targeting AHSA1 in cancer treatment [18, 21, 22].

In present study, we explored the function of AHSA1 in MM cell proliferation and PI resistance, revealed the mechanism on how Bufalin targeted AHSA1 to suppress MM development, and identified the potential therapeutic effects of KU-177 as a selective AHSA1 inhibitor [23].

## Methods

### Gene expression profiling (GEP)

GEP cohorts were collected using the GEO database as previously described [24, 25]. The total therapy 2 (TT2), the Dutch-Belgian Cooperative Trial Group for Hematology Oncology Group-65 (HOVON65) trial patient cohort and the Assessment of Proteasome Inhibition for Extending Remission (APEX) patient cohort were included in these analyses, which utilized publicly available gene expression profile data for each patient cohort [26–28].

### Antibodies and reagents

Antibodies were as follows: AHSA1 (83036, Abcam, UK); HSP90 (13171-1-AP, ProteinTech Group, China); PSMD2 (14748-1-AP, ProteinTech Group, China); CDK6 (14052-1-AP, ProteinTech Group, China); HA (51064-2-AP, ProteinTech Group, China); MYC (16286-1-AP, ProteinTech Group, China); FLAG (F-4020, Merck KGaA, Germany); GAPDH (60004-1-Ig, ProteinTech Group, China); PARP (9542S, Cell Signaling Technology, USA); Caspase-3 (9662S, Cell Signaling Technology, USA); β-actin (4970S, Cell Signaling Technology, USA); Rabbit IgG (a7016, Beyotime Institute of Biotechnology, China) and mouse IgG (a7028, Beyotime Institute of Biotechnology, China).

Doxycycline (DOX) was purchased from the Beyotime Institute of Biotechnology (Shanghai, China). Puromycin was purchased from Merck KGaA (Darmstadt, Germany). Bortezomib (BTZ) and Adriamycin (ADR) were purchased from Selleck Chemicals (Houston, TX). KU-177 was synthesized and characterized in Dr. Nianguang Li’s lab. Carfilzomib (CZ) was obtained from APExBIO Technology LLC (Houston, TX, US).

### Cell lines and cell culture

Human MM cell lines ARP1 and H929, and mouse MM cell line 5TMM3VT were cultured in RPMI-1640 (Biological Industries, Israel). HEK293 cells were cultured in DMEM (Thermo Fisher Scientific, USA). ANBL6 cells were also cultured in RPMI-1640 and BTZ-resistant (BR) capacity was generated by long-term BTZ selection. Flow MRD-positive cells were collected from peripheral blood of newly diagnosed and relapsed MM patients in the Nanjing Drum Tower Hospital, the Affiliated Hospital of Nanjing University of Chinese Medicine. The culture medium was supplemented with 10% fetal bovine serum (Gibco, USA), 100 U/mL penicillin, and 100 μg/mL streptomycin (HyClone, USA). All cells were cultured at 37°C in 5% CO_2_ incubator.

### Plasmids and transfection

The plasmids containing human AHSA1 cDNA and AHSA1 shRNA cassettes were purchased from Generay Biotech Co., (Shanghai, China). The AHSA1 coding sequence was cloned into a lentiviral vector, CD513B-1. AHSA1-targeting shRNA under the control of a DOX-inducible promoter was cloned into the pTRIPZ vector. Lentiviruses were produced by co-transfection of the expression vector of interest with the packaging plasmids PLP1, PLP2, and VSVG into HEK293 cells using Hieff Trans™ Liposomal Transfection Reagent (Cat#40802, Yeasen, China). Virus supernatant was collected at 48 h. Transfected MM cells were selected by puromycin resistance. Transduction efficiency was determined by western blotting (WB).

### Cell proliferation, colony formation, and cell cycle assays

Cell proliferation was determined by MTT assay. Briefly, the cells were seeded into 96-well plates (6×10^3^ cells per well). Then, 20 μL of MTT reagent (5 mg/mL; Sigma-Aldrich, USA) was added into each well after incubated for 24, 48 or 72 h. The formazan was dissolved in DMSO and the absorbance was measured spectrophotometrically at 570 nm by a microplate reader (Thermo Fisher, USA).

For colony formation assays, clonogenic growth was determined by plating 1 x 10^4^ cells in 0.5 mL of 0.33% agar/RPMI 1640 supplemented with 10% FBS. The culture medium was changed twice weekly, and the cells were cultured for around 14 days. Clusters of cells were considered to be a clonogenic colony if >40 cells were present. The colonies were imaged, and colony numbers were counted using ImageJ.

For cell cycle assays, MM cells were fixed using 70% ethanol and washed with PBS and treated with propidium iodide (PI) solution (Yeasen, China) for 30 min. The cell samples were analyzed using flow cytometry (Merck Millipore, Germany).

Annexin V/PI staining assay was used to detect apoptosis of cells. The cells were harvested and resuspended in 100 μL of binding buffer and stained with 5 μL of APC Annexin V (Biolegend, USA) and PI (Solarbio, China) in darkness at room temperature for 15 min. Flow cytometry was performed by Guavaeasy Cyte (Merck Millipore, USA) and Annexin V positive cells were quantitated.

### WB and co-immunoprecipitation (Co-IP)

WB was performed as previously described [29]. Co-IP was conducted by using a Pierce Direct Magnetic IP/Co-IP kit (Thermo Scientific) in accordance with the manufacturer’s instruction.

### Mass spectrometry (MS) analysis

SDS-PAGE was used to separate proteins, and gel bands were excised and digested with sequencing-grade trypsin (Promega, USA). The resulting peptides were analyzed using a QExactive mass spectrometer (Thermo Fisher Scientific). Fragment spectra were analyzed according to the National Center for Biotechnology Information nonredundant protein database.

### Construction of hHsp90α-hAHSA1 structure

The crystal structures of hHsp90β (PDB code: 5FWK [30]) and yAHSA1 (PDB code: 1USU [31]) were retrieved from Protein Data Bank (https://www.rcsb.org/) and prepared by the Molecular Operating Environment (MOE) software (Chemical Computing Group, version 2014.0901, Inc.: Montreal, Canada). The sequences of hHsp90α and hAHSA1 were obtained from UniProt [32] and aligned to the crystal structure sequences of hHsp90β and yAHSA1, respectively. A three-dimensional model of hHsp90α was constructed using the MOE software based on the prepared crystal structure of hHsp90β, and used as “Induced Fit” atoms when building hAHSA1 model using yAHSA1 as a template. Amber99 [33] was selected as force field, and an optimal structure was refined and selected based on the best-scoring intermediate model.

### Small molecule-AHSA1 binding mode prediction

The complex model was used to perform binding site prediction using Schrodinger SiteMap [34, 35] algorithm (version 4.6.011). We mainly focused on the cavity which was composed by hHsp90 and hAHSA1. The corresponding glide docking grids were prepared using defaults. The small molecule ligands (in SMILES format) preparation calculations were performed with LigPre panel in Maestro 11.5. The most energetically favorable conformations were docked using Glide [36] (version 78011) XP (extra precision). In order to investigate detailed binding information between KU-177 and hAHSA1, we performed more computational cost Induced Fit docking. The binding mode between Bufalin and hAHSA1 was manually built and refined by MOE.

### MM Murine models

All animal studies were conducted in accordance with the Government-published recommendations for the Care and Use of Laboratory Animals, and they were approved by the Institutional Ethics Review Boards of Nanjing University of Chinese Medicine (Ethics Registration no. 201905A003).

### MM xenografts

1 x 10^6^ wild type (WT) and AHSA1-overexpression (AHSA1-OE) cells were injected subcutaneously into the left and right abdominal flanks of 6-8 weeks old SCID/NOD mice, respectively. Then, the mice were treated with intraperitoneal (IP) administration of BTZ (1 mg/kg) or ADR (1 mg/kg) twice weekly [37–39]. Tumor diameter was measured 6-7 times weekly using calipers. Once the tumor diameter reached 20 mm, the mice would be sacrificed, then tumor tissues were collected, weighed, and photographed.

### 5TMM3VT mouse model

5TMM3VT mouse myeloma cells (1 x 10^6^) were injected intravenously into the tail vein of 6-week old C57BL/KaLwrij mice. After 2 days, KU-177 and BTZ were intraperitoneally injected at the dose of 1mg/kg twice a week till the mice were sacrificed or dead. The mice would be sacrificed once they exhibited signs of hindlimb weakness. The survival time of the mice in each group was recorded. In another experiment, the 5TMMVT mouse model was replicated, and the toxicity of KU-177 treatment for 4 weeks was evaluated in the main organs.

### Hematoxylin eosin (HE) staining

HE staining was performed on 3 μm paraffin tissue sections mounted on APES-coated slides. The main process was as follows: The slides were conventional dewaxing and rehydration. The tissues were stained with Hematoxylin for 5 min. After washed with distilled water, the tissues were stained with eosin for 10 min. Then the slides were washed with distilled water again, and the tissues were dehydrated and sealed neutral gum.

### Immunohistochemistry analysis (IHC)

IHC staining was performed on 3 μm paraffin tissue sections mounted on APES-coated slides. The main process was as follows: The slides were incubated with the primary antibody overnight at 4°C. Afterwards, the secondary antibody was applied and kept for 45 min at 37°C, followed by dropping SABC at 37°C for 30 min, DAB coloring, and finally counterstaining with hematoxylin.

### Statistical analyses

Statistical analyses were performed using SPSS version 22.0 or GraphPad Prism 6.01 software, and all values were expressed as mean ± SD unless otherwise specified. A two-tailed Student’s t-test (2 groups) or one-way analysis of variance (ANOVA) with Tukey’s post-hoc comparison (≥3 groups) was utilized to evaluate statistical significance. A Kaplan–Meier curve and Log-rank test were employed to determine MM patient survival. *p*<0.05 was considered statistically significant.

## Results

### Elevated AHSA1 expression indicates poor outcomes in MM patients and promotes MM cell proliferation

**Fig. 1:**
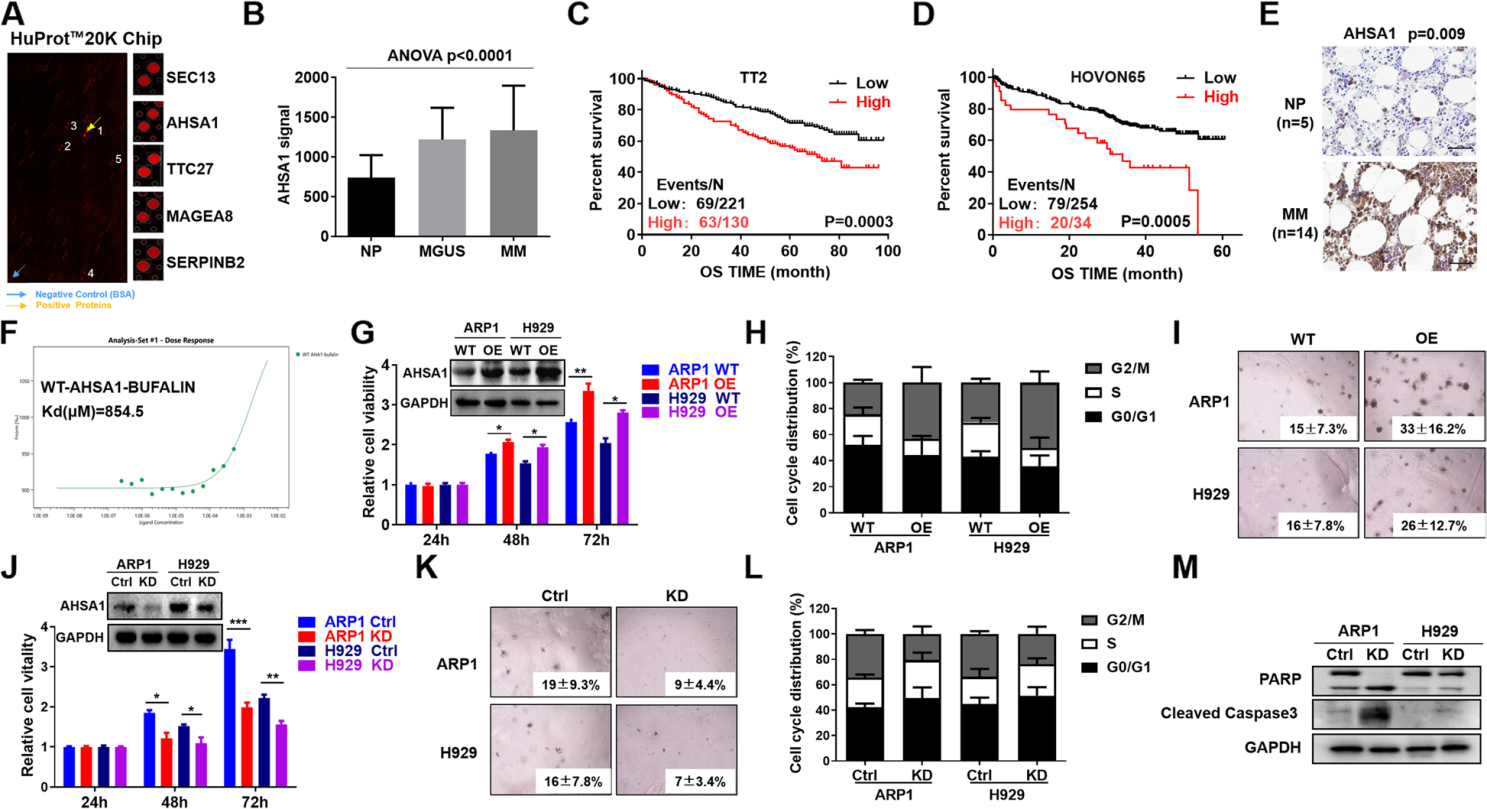
Elevated AHSA1 expression confers poor survival of MM patients and promotes MM cell proliferation. **A** HuProt™20K Proteome Microarray Chip indicated the top 5 protein targets binding to Bufalin. The yellow arrow indicated positive protein interacted with Bufalin, and the blue arrow represented the negative control. **B** Among the top 5 proteins, AHSA1 was the exclusive gene. The signal level of AHSA1 was shown on the y-axis. Patients designated as healthy donors with normal bone marrow plasma cells (NP, *n*=22), monoclonal gammopathy of undetermined significance (MGUS, *n*=44) or multiple myeloma (MM, *n* = 351) were sorted on the x-axis. **C-D** Increased AHSA1 mRNA expression was positively associated with poor overall survival (OS) in first diagnosis and relapsed MM patients from (**C**) TT2 and (**D**) HOVON65 patient cohort. Events/N means events of death/total patients. **E** Representative Immunohistochemistry staining on primary MM samples (*n*=14) and normal controls (*n* = 5). **F** Microscale thermophoresis (MST) analysis for the interaction of Bufalin with human AHSA1 recombination protein. **G** Validation of AHSA1 overexpression in AHSA1*-*OE ARP1 and H929 cells relative to control cells. **H** Cell cycle analysis for WT and AHSA1-OE cells. **I** Representative images of cell colonies of WT and AHSA1-OE cells in soft agar. **J** Confirmation of AHSA1 protein knockdown in ARP1 and H929 cells after transfection with AHSA1 shRNA. **K** Representative images of cell colonies of WT and AHSA1-KD cells in soft agar. **L** Cell cycle analysis for WT and AHSA1-KD cells. **M** WB analysis of PARP and Caspase 3. The data are expressed as mean ± SD.*p<0.05, **p<0.01, ***p<0.001

To explore the potential therapeutic target of Bufalin in MM, we labeled Bufalin with Cy5, a fluorescent probe, then performed HuProt™20K Proteome Microarray Chip to detect the protein target binding to Bufalin. 428 proteins showed significant interaction with Bufalin on the chip (Fig. 1A). We screened all the targets by using the GEP cohorts of MM patients. Among the top 5 targets, only AHSA1 expression was increased in MM samples compared to the normal plasma cells (Fig. 1B) and significantly associated with poor outcomes of MM patients in both TT2 (GSE2658) and HOVON65 (GSE19784) cohorts (Fig. 1C & D). In agreement of above results, IHC assay showed that AHSA1 strongly expressed in MM primary samples relative to the normal control tissues (Fig. 1E). Furthermore, microscale thermophoresis (MST) analysis displayed that Bufalin evidently interacted with human AHSA1 recombination protein (Fig. 1F). Here we inferred AHSA1 might be a novel target of Bufalin in MM. Therefore, we further explored the function of AHSA1 in MM cells. Initially, we forced AHSA1 expression in ARP1 and H929 MM cell lines by lentivirus system. Western blot confirmed the overexpression efficiency and MTT assay showed that elevation of AHSA1 promoted MM cell growth (Fig. 1G). AHSA1-OE cells were characterized by increased fraction of G2/M phase in cell cycle compared to WT cells (Fig. 1H). In addition, AHSA1-OE cells also showed increased long-term cell growth by colony formation assay compared to WT cells (Fig. 1I). Inversely, knockdown of AHAS1 expression by shRNA abrogated these features in cells growth (Fig. 1J & K) and cell cycle (Fig. 1L), and induced MM cell apoptosis (Fig. 1M).

### AHSA1 is a MM high-risk marker and induces proteasome inhibitor resistance *in vitro* and *in vivo*

**Fig. 2:**
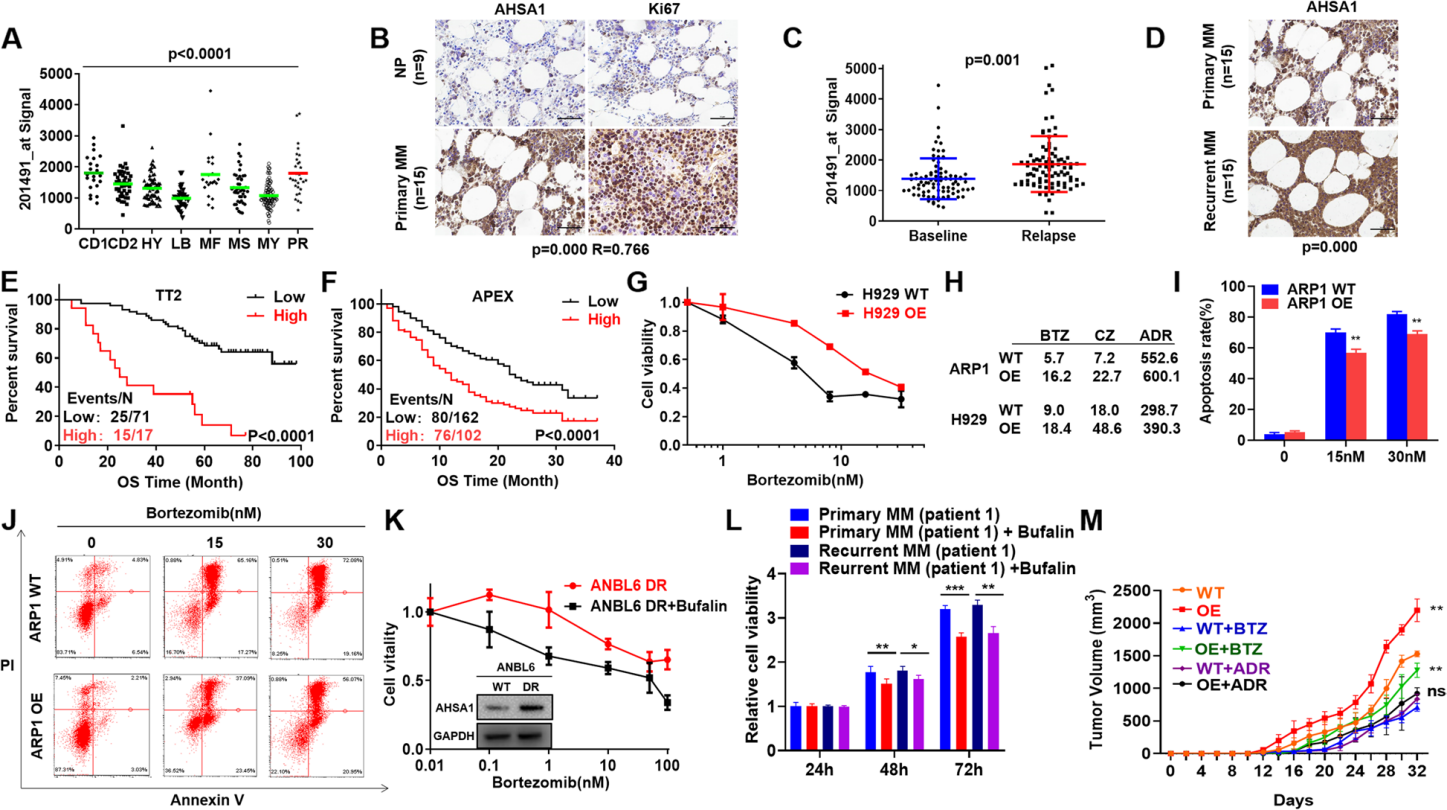
AHSA1 is a high-risk MM marker and induces proteasome inhibitor resistance *in vitro* and *in vivo*. **A** Box plot representing AHSA1 expression in eight MM risk subgroups from TT2 patient cohort. **B** IHC staining of AHSA1 and Ki67 expressions in MM patient samples. **C** AHSA1 mRNA expression in paired patient MM samples collected at first diagnosis and relapse stage. **D** IHC staining of AHSA1 expression in the relapsed samples and the corresponding samples from first diagnosis. **E-F** Elevated AHSA1 expression was correlated with decreased OS in relapsed patients from the (**E**) TT2 and (**F**) APEX cohorts by long-term following up. **G** Effects of Bortezomib on cell viability of H929 cells with or without overexpression of AHSA1. **H** IC_50_ values of BTZ, CZ and ADR in MM cells with or without overexpression of AHSA1. **I** The rate of BTZ-induced apoptosis was shown in the histogram. **J** Effects of BTZ on cell apoptosis in ARP1 cells with or without overexpression of AHSA1. **K** Effects of Bufalin on cell viability in ANBL6 DR (Bortezomib-resistant) cells. **L** Effects of Bufalin (60nM) on the cell viability of flow MRD-positive peripheral cells from first diagnosed and relapsed MM patients. **M** Time course of tumor growth in ARP1 AHSA1 WT/OE xenografts taken from NOD-SCID mice treated with vehicle, BTZ, or ADR. The data are expressed as mean ± SD.*p<0.05, **p<0.01, ***p<0.001

We further detected the distribution of AHSA1 in the molecular classification of MM subgroups [24]. AHSA1 exhibited the highest expression in PR subgroup, the worst and high-risk subgroup featured by high proliferation in MM (Fig. 2A). Consistently, the similar positive association between AHSA1 and MM cell proliferation was observed by performing IHC in MM primary tissues with application of Ki67, a marker of proliferation (Fig. 2B). As most of MM patients eventually developed relapse, we also tested AHSA1 expression in relapsed MM samples. As shown in Fig. 2C, AHSA1 expression was increased in 88 paired MM samples collected at relapse and newly-diagnose stage of the same patients, which was consistent with the result of IHC staining at protein level (Fig. 2D). Intriguingly, AHSA1 expression was significantly associated with poor outcome of MM relapse patients in TT2 by long-term following up (GSE31161) and APEX cohorts (Fig. 2E & F). Since relapse MM patients usually acquired drug resistance, we continued to detect if AHSA1 was related to drug resistance in MM patients. The IC_50_ of three classic chemo-drugs for MM treatment, Bortezomib (BTZ), Carfilzomib (CZ) and Doxorubicin (ADR), was tested by MTT assay. Overexpression of AHSA1 induced proteasome inhibitor resistance, while there was no bias to ADR (Fig. 2G & H). Apoptosis analysis also supported above result indicating that striking anti-apoptosis effect was observed in AHSA1-OE cells relative to WT cells (Fig. 2I & J, Supplementary Fig. 1A & B). Consistently, AHSA1 expression was higher in ANBL6 BTZ-resistant cells than ANBL6 WT cells, while Bufalin abrogated the BTZ resistance induced by AHSA1 and inhibited cellular growth of both WT and BTZ-resistant cells (Fig. 2K). In addition, Bufalin treatment hampered the proliferation of flow MRD-positive cells in both primary and recurrent MM patient samples (Fig. 2L & Supplementary Fig. 1C). Finally, MM xenograft model confirmed above findings *in vivo* and demonstrated that upregulation of AHSA1 promoted MM cell growth and BTZ resistance *in vivo* (Fig. 2M & Supplementary Fig. 1D-F).

### AHSA1 promotes MM cell proliferation and BTZ resistance through activating CDK6 and PSMD2, respectively

**Fig. 3:**
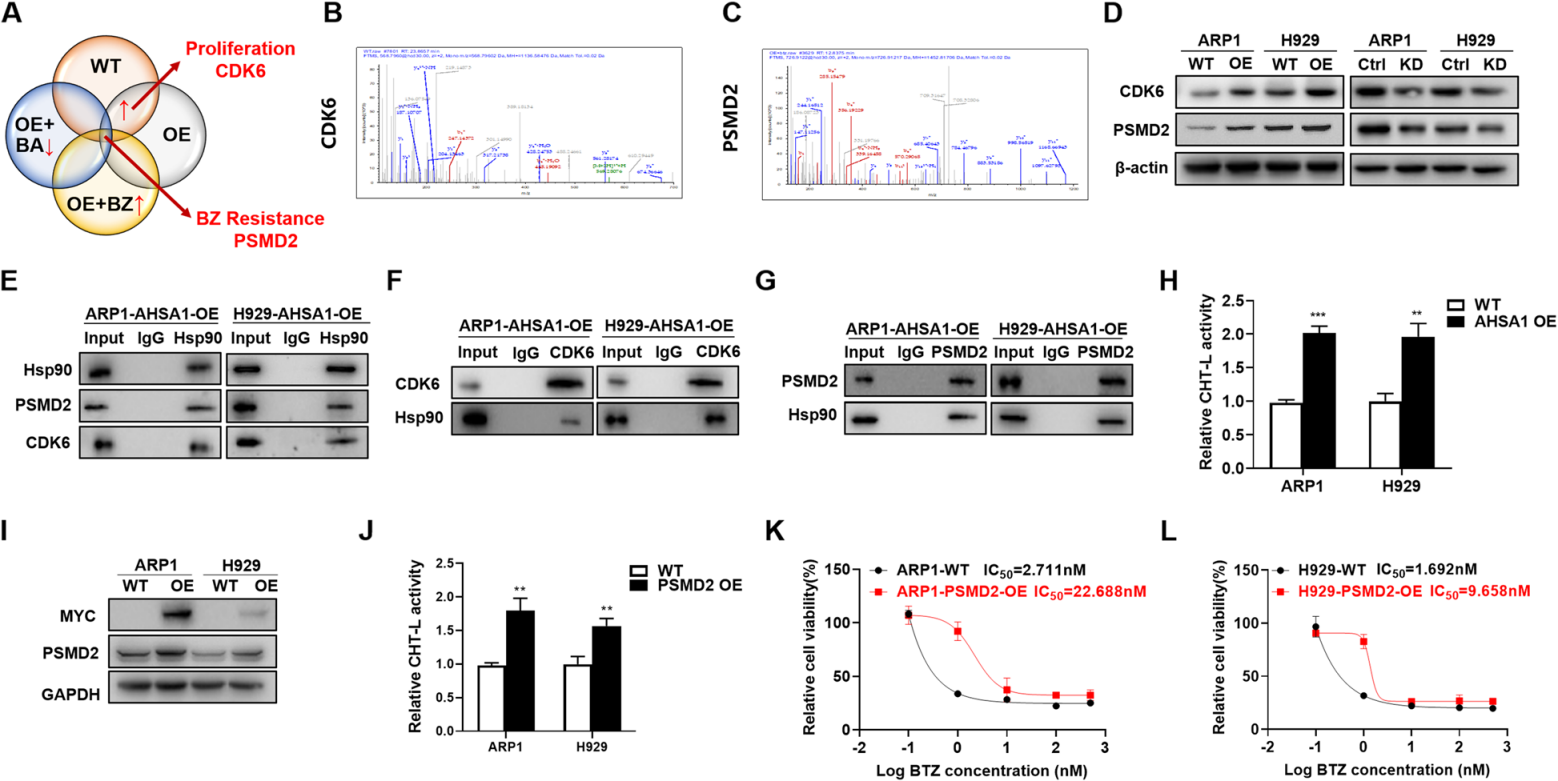
AHSA1 promotes MM proliferation and BTZ resistance through activating CDK6 and PSMD2 respectively. **A-C** Using Co-IP assay followed by MS, CDK6 and PSMD2 were selected among candidate genes of the proliferation-related and drug-resistance genes in ARP1 WT, AHSA1-OE cells, AHSA1-OE cells treated with Bufalin or BTZ, respectively. **D** WB analysis showed the expressions of CDK6 and PSMD2 in AHSA1-OE (Left) and AHSA1-KD cells (Right). **E** Co-IP experiment further confirmed the interaction between HSP90 with CDK6 and PSMD2 in ARP1 and H929 cells using HSP90 antibody as bait. **F** Co-IP experiment showed that CDK6 directly interacted with HSP90 in ARP1 and H929 cells. **G** Co-IP assay confirmed that PSMD2 interacted with HSP90 in ARP1 and H929 cells. **H** Proteasome activity assay showed that overexpression of AHSA1 in ARP1 and H929 cells resulting in high proteasome activity. **I** Validation of PSMD2 overexpression in ARP1 and H929 PSMD2-OE cells relative to WT cells. **J** Proteasome activity assay showed that overexpression of PSMD2 in ARP1 and H929 cells led to high proteasome activity. **K-L** Effects of BTZ on the cell viability of ARP1 (**K**) and H929 (**L**) cells with or without PSMD2 overexpression. The data are expressed as mean ± SD.*p<0.05, **p<0.01, ***p<0.001

To identify the mechanism on how AHSA1/HSP90 complex promoted MM cell proliferation and BTZ resistance, we performed a Co-IP assay followed by MS method to determine the proteins interacting with HSP90 in ARP1 WT, AHSA1-OE cells, AHSA1-OE cells treated with Bufalin or BTZ. The abundance of proteins promoting cellular growth was increased in AHSA1-OE cells relative to WT cells tested by Co-IP/MS methods, and CDK6, a key proliferative factor [37, 40, 41], was in the candidate list (Fig. 3A & B). Using the similar strategy, we screened out PSMD2 (26S proteasome non-ATPase regulatory subunit 2) as the BTZ-resistant candidate protein [42], while Co-IP/MS tests validated that its abundance was increased in AHSA1-OE cells and AHSA1-OE cells treated with BTZ, but decreased in AHSA1-OE cells with Bufalin treatment compared to WT cells (Fig. 3A & C). WB analysis showed that increased AHSA1 upregulated CDK6 and PSMD2 expression, in contrast decreased AHSA1 downregulated both two factors correspondingly in MM cells (Fig. 3D). Co-IP assay further confirmed the interaction between HSP90 with CDK6 and PSMD2 in ARP1 and H929 cells using HSP90 antibody as bait (Fig. 3E). In turn, HSP90 was blotted in these cells using CDK6 as bait (Fig. 3F) that indicated CDK6 was a client protein of AHSA1/HSP90 to promote MM cell growth.

Similarly, HSP90 was also co-immunoprecipitated by PSMD2 antibody in MM cells (Fig. 3G). Since the upregulation of 26S proteasome subunits could induce PI resistance [42, 43] by elevating the proteasome activity, we first validated that overexpression of AHSA1 promoted the ChT-L activity of proteasome in MM cells (Fig. 3H). Then we verified that increased expression of PSMD2 in MM cells upregulated the ChT-L activity of proteasome (Fig. 3I & J) and exerted PI-resistant activity (Fig. 3K & L). Now we may conclude that AHSA1 promotes BTZ resistance via activating PSMD2 in MM.

### Bufalin decreases cellular proliferation and PI resistance induced by AHSA1/HSP90 in MM

**Fig. 4:**
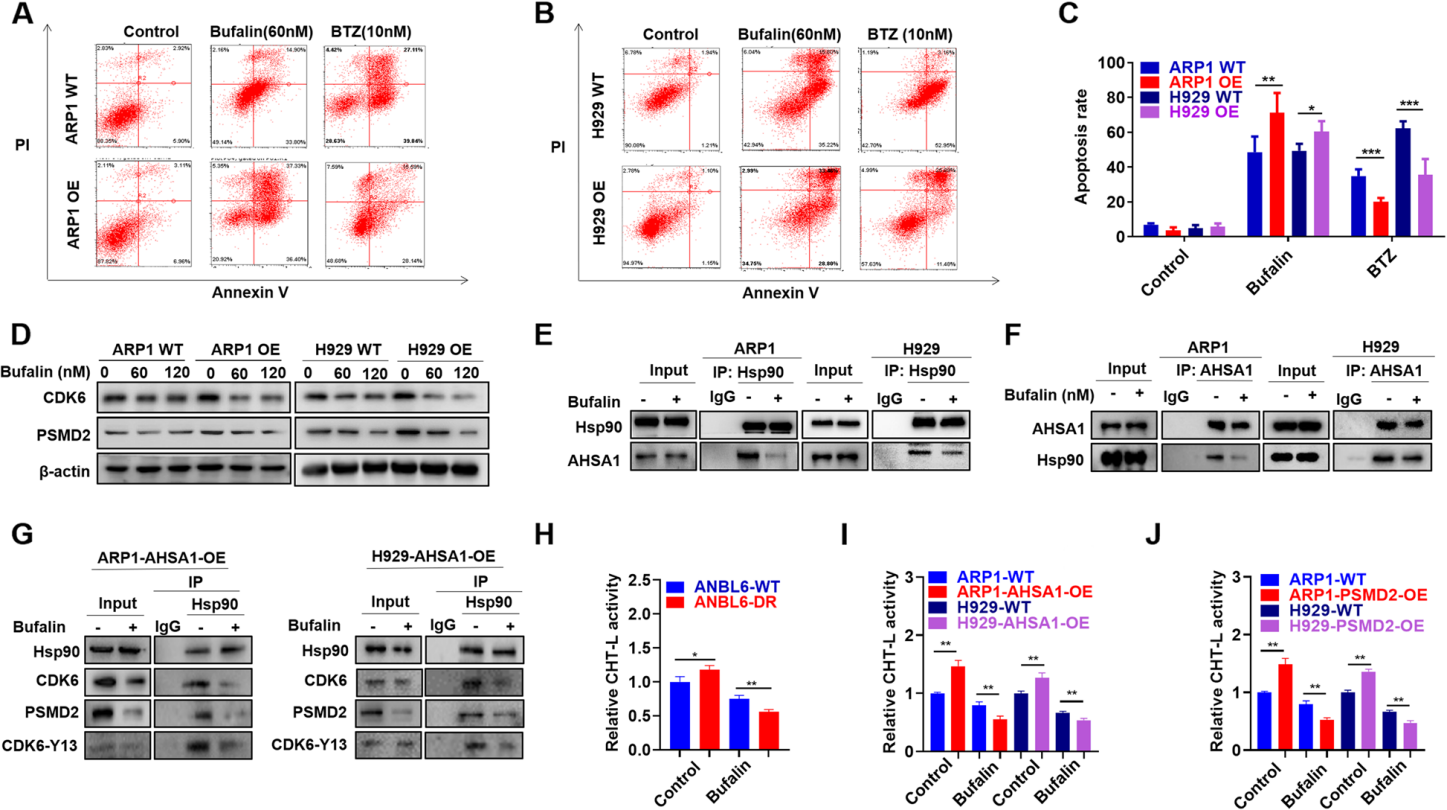
Bufalin decreases cellular proliferation and PI resistance induced by AHSA1/HSP90 in MM cells. **A-B** Effects of Bufalin (60nM) and BTZ (10nM) incubation for 48h on cell apoptosis of ARP1 (**A**) and H929 (**B**) WT and AHSA1-OE cells. **C** The rate of drug-induced apoptosis was shown in the histogram. **D** Effects of Bufalin on the expression of CDK6 and PSMD2 in ARP1 and H929 WT and AHSA1-OE cells. **E-F** Co-IP assay revealed that Bufalin interfered the interaction between HSP90 and AHSA1 in ARP1 and H929 cells. **G** Co-IP assay confirmed the interaction between AHSA1, HSP90, CDK6, PSMD2 and the activated form of CDK6, phosphorylation of Y13 site at CDK6. **H-J** Proteasome activity assay verified that Bufalin inhibited proteasome activity in (**H**) ANBL6 WT/DR cells, **I** ARP1 and H929 AHSA1 WT/OE cells and **J** PSMD2 WT/OE cells. The data are expressed as mean ± SD.*p<0.05, **p<0.01, ***p<0.001

To test if Bufalin could decrease MM cell proliferation and PI resistance induced by AHSA1/HSP90, we performed Annexin V/PI staining assay in MM cells treated with BTZ and Bufalin. As shown in Fig. 4A-C, AHSA1-OE cells were significantly resistant to BTZ-induced cellular apoptosis compared with WT cells, while Bufalin treatment could reverse PI resistance caused by overexpressed AHSA1. Next, western blot analysis demonstrated that Bufalin decreased MM cell proliferation and PI resistance by reducing CDK6 and PSMD2 expression in both AHSA1 WT/OE cells (Fig. 4D). To identify how Bufalin mediated CDK6 and PSMD2, we performed Co-IP assay using AHSA1-OE cells treated with or without Bufalin, and found that Bufalin interfered the interaction between AHSA1 and HSP90 utilizing each antibody as bait mutually (Fig. 4E & F). We also confirmed that interfering the interaction between AHSA1 and HSP90 by Bufalin decreased the expression of CDK6 and PSMD2, the AHSA1/HSP90 client proteins (Fig. 4G). In addition, the activated form of CDK6, phosphorylation of Y13 site at CDK6 [44], was also abated by Bufalin treatment (Fig. 4G). We further proved the effect of Bufalin on PI resistance and corroborated Bufalin inhibiting the ChT-L activity of proteasome in ANBL6 WT/DR cells (Fig. 4H), AHSA1 WT/OE cells (Fig. 4I) and PSMD2 WT/OE cells (Fig. 4J).

### AHSA1-K137 is identified as the site-specific targeting of Bufalin and KU-177

**Fig. 5:**
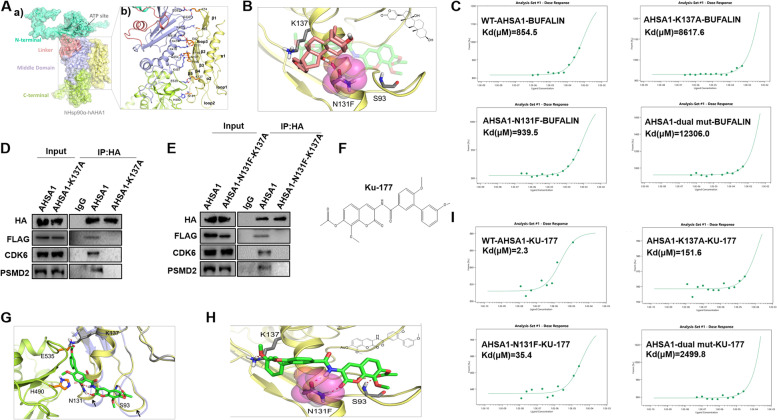
AHSA1-K137 is identified as the site-specific targeting of Bufalin and KU-177. **A** hHsp90α-hAHSA1 complex model. **a** Overview of hHsp90α (left) interacted with hAHSA1 (right, colored in yellow). Both hHsp90α and hAHSA1 were represented in colored cartoon and corresponding transparent surface. The N-terminal (15-287), linker (288-323), middle domain (324-476), and C-terminal (477-698) domain of hHsp90α were colored in cyan, red, blue and green, respectively. **b** Detailed information of hHsp90α binding with hAHSA1. The key residues corresponding to hAHSA1 bound with hHsp90α were shown as orange and blue sticks, respectively. The key interactions between residues were depicted by red dotted lines. **B** Predicted binding modes of Bufalin targeting hAHSA1. Bufalin was shown in red sticks, and hAHSA1 was shown in yellow cartoon. Key residues were shown in gray sticks, and hydrogen bonds were depicted by dotted lines. Phenylalanine in mutation of AHSA1-N131F was shown in magenta sphere. **C** MST results of Bufalin on wild and site-directed mutagenesis (SDM) of hAHSA1. AHSA1-K137 was involved in hydrogen bond interactions with Bufalin, and this hydrogen bond interaction disappeared with mutation to alanine. **D-E** AHSA1-K137 was involved in hydrogen bond interactions with its co-chaperone, HSP90, and AHSA1/HSP90 client protein, CDK6 and PSMD2, and this hydrogen bond interaction disappeared while mutation to alanine or double mutation K137A/N131F, as demonstrated by Co-IP using HA antibody as bait in ARP1 HSP90-OE cells followed by WB. AHSA1-OE plasmid was linked with HA tag, while HSP90-OE plasmid was linked with FLAG tag. **F** The chemical structure of KU-177. **G** Structural details of the predicted binding modes of KU-177. KU-177 was shown in green sticks, and key residues of hHsp90α (green cartoon) and AHSA1 (yellow cartoon) were shown in orange and gray sticks, respectively. Conformational changes from a hHsp90α-binding conformation (blue transparent cartoon) to a KU-177 “Induced Fit” conformation (yellow cartoon), as well as corresponding residues, K137 and N131, were also presented. **H** Predicted binding modes of KU-177 targeting hAHSA1. KU-177 was shown in green sticks, and hAHSA1 was shown in yellow cartoon. Key residues were shown in gray sticks, and hydrogen bonds were depicted by dotted lines. Phenylalanine in mutation of N131F was indicated in magenta sphere. **I** MST results of KU-177 on wild and site-directed mutagenesis (SDM) of hAHSA1. K137 was involved in hydrogen bond interactions with KU-177, and this hydrogen bond interaction would disappear with mutation to alanine

We established hHsp90α-AHSA1 binding model based on hHsp90β (PDB code: 5FWK [45], shared 94.5% sequence similarity with hHsp90α) and AHSA1 (PDB code: 1USU [31]) structures to further explore the potential targeting site of AHSA1 with Bufalin. As shown in Fig. 5A, Hsp90α was well-conserved but highly dynamic [46, 47], and each hHsp90 monomer contained four structural domains (Fig. 5A, a): a N-terminal domain (NTD), a linker, a middle domain (MD) and a C-terminal domain (CTD). The NTD of hAHSA1 contained 2 α-helixes, 5 β-sheets and 3 loops (Fig. 5A, b). The interfaces of two proteins were relatively open and polar which might form extensive network of solvent bridges, and a complementary pattern of charged patches on the opposing surfaces. The core of the interactions with AHSA1 was provided by polar residues from hHsp90α. Higher up on the first domain of MD of hHsp90α, D393 and G333 on the exposed loop region interacted with K74 from hAHSA1. Polar residues K407, K414, N415 and K418 of the helical coil segment of the hHSP90α MD formed an extensive network of hydrogen bonding and ion-pair interactions with S69, E82 and E111 of β-sheet section on hAHSA1. The lower loop segment of hHsp90α MD interacted via hydrogen-bond/ionic interaction involving Q454 and K457 from hHsp90α, and P106, N107 and E116 of loo3 of AHSA1. Apart from the MD, CTD (E535 and H490) of hHsp90α also could form interactions with α2 helix of hAHSA1 (K137 and N131).

Next, molecular modeling study revealed that the main interaction between Bufalin and hAHSA1 was located at a hydrogen bond linking the carbonyl with K137. Additionally, another hydrogen bond appeared between the hydroxyl group and the backbone of N131 (Fig. 5B). Mutation of K137A led to a 10.1-fold reduction in affinity of Bufalin (Fig. 5C). Moreover, double mutations of K137A/N131F resulted in 14.4-fold decrease in affinity of Bufalin (Fig. 5C). Consistently, AHSA1-K137 was involved in hydrogen bond interactions with its co-chaperone HSP90α and AHSA1/HSP90 client proteins CDK6 and PSMD2. These hydrogen bond interactions disappeared when mutation of alanine or double mutation K137A/N131F was applied, as described by Co-IP method (Fig. 5D-E).

Due to severe side-effects and toxicities of Bufalin [12, 48] which limited its application for clinical treatment, we synthetized the selective inhibitor KU-177 of AHSA1, the target of Bufalin [23] (Fig. 5F). As shown in Fig. 5G, KU-177 exclusively bound to the α2 helix and loop2 region of hAHSA1. The main interactions between KU-177 and hAHSA1 was located between: i) methoxy group interacted with K137; ii) the carbonyl of coumarin and the amide group contacted with N131 through two hydrogen bonds; iii) the acetyl group formed a hydrogen bond with S93. Next, Mutation of K137A, N131F resulted in a 65.9-fold and 15.4-fold decrease in affinity of KU-177 evaluated in hAHSA1 studies, respectively. The double mutation of K137A/N131F caused a significant 1086.9-fold reduction in affinity of KU-177.

As indicated in Fig. 5B, C, G, H, I, K137 was involved in hydrogen bond interactions with both KU-177 and Bufalin, and this hydrogen bond interaction would disappear with mutation of alanine, resulting in a decrease in affinity of both compounds. In addition, mutation of N131F also showed a moderated decrease in affinity, for the disappear of hydrogen bonds between small molecules and N131, as well as steric and conformational compatibility (Fig. 5B and H).

KU-177 and Bufalin were bound to AHSA1 through interacting with key residue (K137 and N131) and occupied corresponding binding site resulting in disturbing the interaction with hHsp90α, which suggested that it was the biologically relevant unit. Moreover, binding to exogenous molecules would induce conformational change of hAHSA1. These data demonstrated how small molecules bound to hAHSA1 and interfered its biological function.

### KU-177 decreases MM cell proliferation and PI resistance induced by AHSA1/HSP90 *in vitro*

**Fig. 6:**
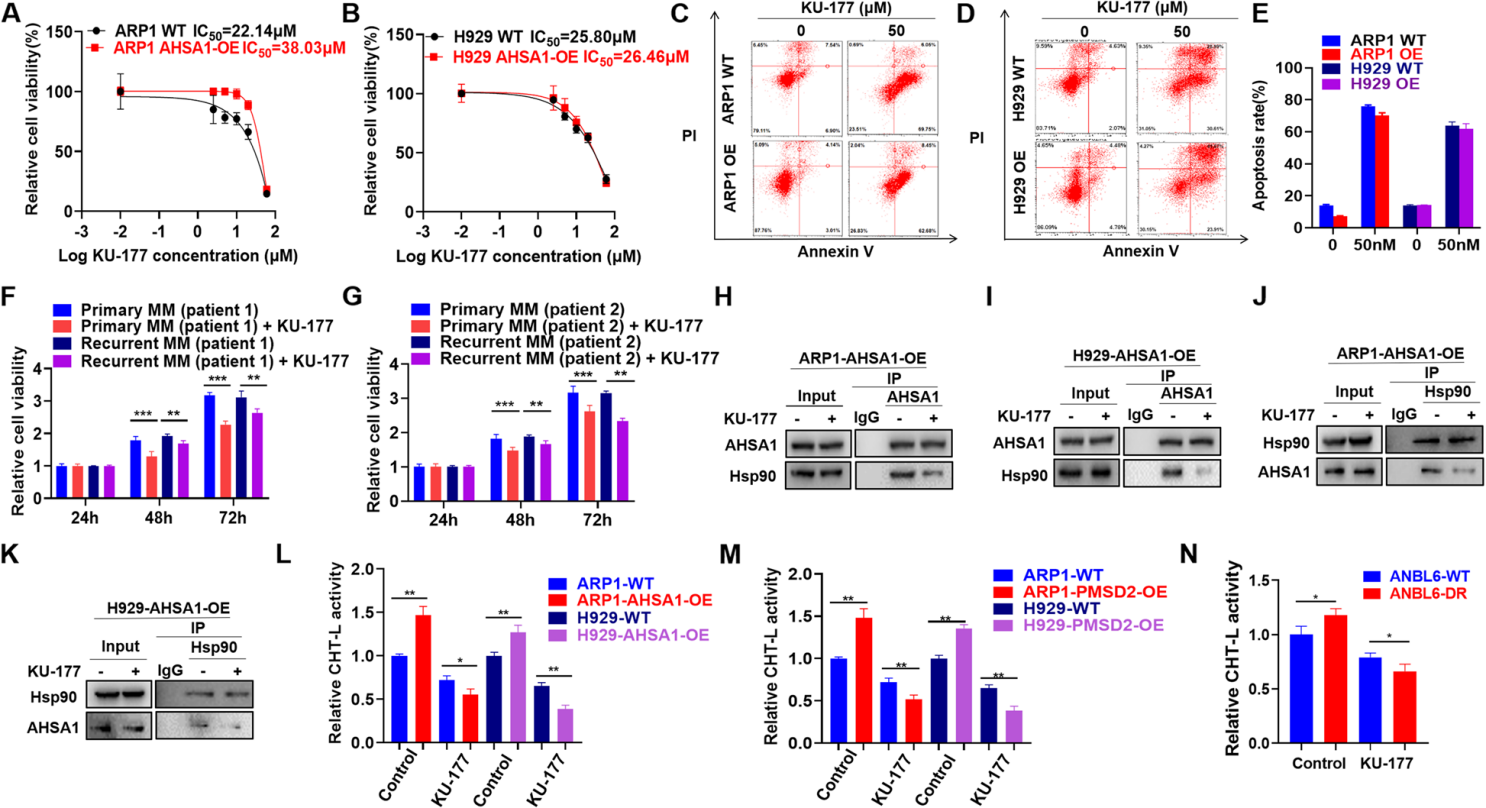
KU-177 decreases MM cell proliferation and PI resistance induced by AHSA1/HSP90 *in vitro*. **A-B** Effects of 48 h treatment with KU-177 on cell viability of ARP1 (**A**) and H929 (**B**) WT and AHSA1-OE cells. **C-E** Effects of 48 h treatment with KU-177 (50 μM) on cell apoptosis of ARP1 (**C**) and H929 (**D**) WT and AHSA1-OE cells. **F** Effects of 48 h treatment with KU-177 (30 μM) on cell viability of flow MRD-positive peripheral cells from first diagnosed and relapsed MM patients. **H-K** Co-IP assay revealed that 48 h treatment with KU-177 (30 μM) inhibited the interaction between HSP90 and AHSA1 in AHSA1-OE cells. **L-N** Proteasome activity assay showed that 48 h treatment of KU-177 (30 μM) inhibited proteasome activity in AHSA1 WT/OE cells (**L**), PSMD2 WT/OE cells (**M**) and ANBL6 WT/DR (**N**) cells. The data are expressed as mean ± SD.*p<0.05, **p<0.01, ***p<0.001. The data are expressed as mean ± SD.*p<0.05, **p<0.01, ***p<0.001

KU-177 inhibited cell growth (Fig. 6A & B) and induced cellular apoptosis in both WT and AHSA1-OE cells (Fig. 6C-E). In addition, MTT assay demonstrated that KU-177 treatment hampered cellular proliferation of flow MRD-positive cells in both primary and recurrent MM patient samples (Fig. 6F-G). Thus, we assumed that Bufalin and KU-177 exerted anti-MM activity in primary patients’ cells. Similar to Bufalin, KU-177 interfered the interaction between AHSA1 and HSP90 in MM cells confirmed by Co-IP assay (Fig. 6H-K). Consistently, KU-177 inhibited the ChT-L activity of proteasome in AHSA1 WT/OE cells (Fig. 6L), PSMD2 WT/OE cells (Fig. 6M) and ANBL6 WT/DR cells (Fig. 6N).

### The combination treatment of KU-177 and BTZ extends the survival of 5TMM3VT MM mice

**Fig. 7:**
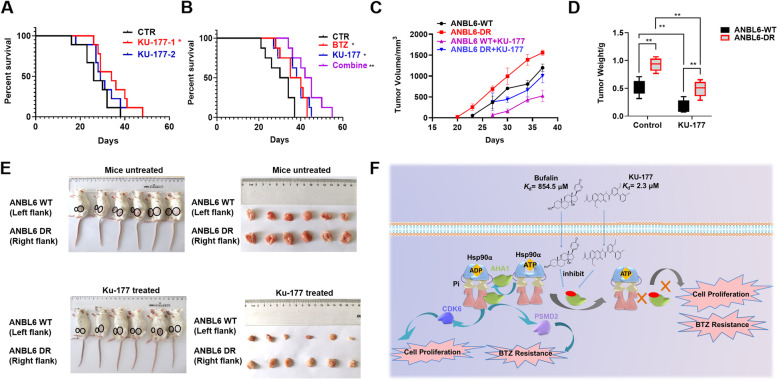
The combination treatment of KU-177 and BTZ extends the survival of 5TMM3VT MM mice. **A** KU-177 greatly extended the survival of 5TMMVT mice. **B** The combination of KU-177 and BTZ significantly improved the survival time of 5TMMVT mice. **C** Time course of tumor growth originated from ANBL6 WT/DR cells in NOD-SCID mice treated with KU-177 or untreated. **D** Mean tumor weight of ANBL6 WT/DR xenografts from KU-177-treated or untreated group at day 37 after injection of MM cells. **E** Photographic images of xenografts in NOD-SCID mice of each group. **F** Graphic working model illustrates that AHSA1 is a promising therapeutic target for cellular proliferation and proteasome inhibitor resistance in MM. The data are expressed as mean ± SD.*p<0.05, **p<0.01, ***p<0.001

Finally, we examined the inhibitory effect of KU-177 on AHSA1 in 5TMM3VT mouse model, which reflected the MM bone marrow microenvironment *in vivo* [49]. At first, we testified the dosage of KU-177 for the treatment of 5TMM3VT mice, and identified that KU-177 at the dosage of 1mg/kg, twice per week could improve the survival outcome significantly relative to 2mg/kg dosage (Fig. 7A). Then we found that the combination treatment of KU-177 (1mg/kg, twice per week) and BTZ (1mg/kg, twice per week) greatly extended the survival of 5TMM3VT mice compared to the control and single treatment group (Fig. 7B). Interestingly, KU-177 also inhibited the xenograft tumor growth of both ANBL6 WT/BTZ-DR cells (Fig. 7C-E). The tumor volume statistics (Fig. 7D) and tumor weight plots (Fig. 7E) demonstrated that KU-177 was potent on both ANBL6 WT/BTZ-DR cells. The microscopic changes of organs were evaluated histologically in different treatment groups. Main organs including heart, liver, spleen, lung and kidney were not observed histopathological abnormities or lesions in Bufalin and KU-177 groups compared with control group (Supplementary Fig. 1G), suggesting that there was no significant toxicity of Bufalin and KU-177 treatments on the mice. In this study, Bufalin and KU-177 were applied as 1mg/kg, twice per week for 4 weeks. These findings indicates that AHSA1 may serve as a novel target for cellular proliferation and PI resistance, and specific AHSH1 inhibitor is promising for the treatment of MM (Fig. 7F).

## Discussion

MM remains an incurable disease, due to the adverse features, such as heterogeneity, acquired drug-resistance and severe malignancy et al. MM patients typically respond to the initial treatment, however most of them eventually developed resistance and relapsed. Our group insists on the discovery of novel diagnostic and therapeutic targets in MM. Primarily, we performed high-throughput screening for novel targets in MM patient microarray and RNA sequencing cohorts [26, 37]. As previously reported, we proved NEK2 acting as a MM drug-resistant gene by comparing 9 pairs sequential MM samples that were collected from the same patients at 4 sequential treatment stages, newly-diagnostic, before the 1^st^ autologous stem cell transplantation (ASCT), before the 2^nd^ ASCT and after the 2^nd^ ASCT stages [50]. We also found that RARalpha2 expression conferred myeloma stem cell features via analyzing paired samples collected from newly diagnose and relapse MM patients respectively [51]. In present study, we further improved our strategy on target screening by combining proteome microarray and microarray cohorts in MM.

Traditional Chinese Medicine (TCM) has made great contribution to the advanced achievements in modern medicine [52, 53], like the discovery of arsenic trioxide on leukemia and Arteannuin on malaria, which were derived from ancient Chinese Medicine records. Inspired by the “like cures like” principle on cancer treatment in Chinese Medicine, we utilized the bioactive anti-cancer molecule Bufalin as a probe, which was originally extracted from Venenum Bufonis, a typical toxic Chinese Medicine to screen out AHSA1 as the potential target in MM by proteome microarray and MM microarray cohorts. Further studies showed that AHSA1 expression was elevated in MM samples compared to normal plasma controls, and significantly associated with MM relapse and poor outcomes. Increased AHSA1 promoted MM cell proliferation and PI resistance *in vitro* and *in vivo* compared to WT cells. Our mechanism exploration disclosed that AHSA1 acted as a co-chaperone of HSP90A to activate CDK6 and PSMD2, which were key regulators of cellular proliferation and PI resistance respectively.

Furthermore, we identified AHSA1-K137 as the specific binding site of Bufalin in AHSA1, mutation of which decreased the interaction of AHSA1 with HSP90A and suppressed the function of AHSA1 on mediating CDK6 and PSMD2. Bufalin possesses severe side-effects and toxicities in animal studies and clinical trials including hematological, gastrointestinal, mucocutaneous and cardiovascular adverse reactions [12, 48] that limit the further utility of Bufalin into clinics. We synthetized an AHSA1 selective inhibitor KU-177 [23] and detected its inhibitory effect on MM cells instead of Bufalin. Intriguingly, KU-177 suppressed cell growth *in vitro*, mediated HSP90 functions and retarded the growth of both WT and PI-resistant MM cells *in vivo*. As KU-177 or other AHSA1 inhibitors may be more specific to AHSA1 compared with Bufalin, we believe that KU-177 has the properties of low toxicity and high efficacy in MM treatment.

As documented, HSP90 has been recognized as a cancer therapeutic target for several decades and over 20 HSP90 inhibitors have entered clinical trials for cancer treatment [19]. However, even now none of the inhibitors was approved eventually for the clinical application due to formulation, off-target, toxicity and cost issues [54]. Our work first demonstrated that AHSA1 was significantly increased in MM samples compared to normal controls and positively associated with MM patients’ outcomes in three independent cohorts including over 1000 patient samples, suggesting that AHSA1 might be a MM-specific target. Furthermore, we inferred that selective inhibition of AHSA1 in MM could attenuate the activity of HSP90 instead of HSP90 inhibitors to avoid side effects. Collectively, our study underlies AHSA1 as a novel target on mediating cellular proliferation and PI resistance in MM, and demonstrates that specific AHSH1 inhibitors have promising prospects in MM treatment.

## Conclusion

Our exploration establishes a strategy to discover novel therapeutic targets in MM from the treasure trove of TCM for bioactive molecules and other effective compounds, which are limited for clinical application as a result of severe side effects. Due to the fact that the chosen probes indeed possess significant bio-activity in cancer treatment, our strategy may be superior to the high-throughput drug screening which consumes a lot of time and finance resources, and greatly reduces the risk for drug development.

## Supplementary Information

**Additional file 1: Figure S1.** AHSA1 induces proteasome inhibitor resistance *in vitro* and *in vivo*. (**A**) Effects of Bortezomib on cell apoptosis in H929 cells with or without overexpression of AHSA1. (**B**) The analysis of bortezomib-induced apoptosis. (**C**) Effects of Bufalin (60nM) on the cell viability in flow MRD-positive peripheral cells from first diagnosed and relapsed MM patients. (**D**) Photographic images of ARP1 AHSA1 WT/OE xenografts taken from NOD-SCID mice treated with vehicle, BTZ, or ADR. (**E**) Mean tumor weight in the six experimental groups at day 32 after implantation of MM cells. (**F**) Western blot analysis of AHSA1 expression of the tumors in the experimental groups. (**G**) Images of representative HE staining of heart, liver, spleen, lung and kidney from control and 5TMM mouse model with or without Bufalin or KU-177 treatment.

## Data Availability

All data that support the findings of this study are available from the corresponding authors upon reasonable request.

## Abbreviations

GEO: Gene expression omnibus
GEP: Gene expression profiling
MS: Mass spectrometry
IF: Immunofluorescence
OS: Overal survival
MM: Multiple myeloma
MGUS: Monoclonal gammopathy of undetermined significance
BM: Bone marrow
TT2: Total therapy 2
PI: Propidium iodide
IHC: Immunohistochemistry analysis
OE: Overexpression
WT: Wild type
TCM: Traditional Chinese Medicine
BTZ: Bortezomib
CZ: Carfilzomib
ADR: Doxorubicin (ADR)
MST: Microscale thermophoresis

## References

[1] van de Donk N, Pawlyn C, Yong KL (2021). Multiple myeloma. Lancet.

[2] Ragoonanan D, Khazal SJ, Abdel-Azim H, McCall D, Cuglievan B, Tambaro FP, et al. Diagnosis, grading and management of toxicities from immunotherapies in children, adolescents and young adults with cancer. Nat Rev Clin Oncol. 2021;7:435–53. 10.1038/s41571-021-00474-4 PMC9393856 33608690

[3] Mohyuddin GR, Rooney A, Balmaceda N, Aziz M, Sborov DW, McClune B, Kumar SK (2021). Chimeric antigen receptor T-cell therapy in multiple myeloma: a systematic review and meta-analysis of 950 patients. Blood Adv.

[4] Kumar SK, Rajkumar V, Kyle RA, van Duin M, Sonneveld P, Mateos MV, Gay F, Anderson KC (2017). Multiple myeloma. Nat Rev Dis Primers.

[5] Cortes J, Perez-Garcia JM, Llombart-Cussac A, Curigliano G, El Saghir NS, Cardoso F, et al. Enhancing global access to cancer medicines. CA Cancer J Clin. 2020;2:105–24. 10.3322/caac.21597 32068901

[6] Siegel RL, Miller KD, Jemal A (2019). Cancer statistics, 2019. CA Cancer J Clin.

[7] Siegel RL, Miller KD, Jemal A (2020). Cancer statistics, 2020. CA Cancer J Clin.

[8] Gu C, Wang W, Tang X, Xu T, Zhang Y, Guo M, Wei R, Wang Y, Jurczyszyn A, Janz S (2021). CHEK1 and circCHEK1_246aa evoke chromosomal instability and induce bone lesion formation in multiple myeloma. Molecular Cancer.

[9] Zhang X, Zhao X, Liu K, Che Y, Qiu X, Qu Y, Sun X, Song J (2020). Bufalin: a systematic review of research hotspots and antitumor mechanisms by text mining and bioinformatics. Am J Chin Med.

[10] Lan YL, Lou JC, Jiang XW, Wang X, Xing JS, Li S, Zhang B (2019). A research update on the anticancer effects of bufalin and its derivatives. Oncol Lett.

[11] Wu XY, Tian F, Su MH, Wu M, Huang Y, Hu LH, Jin L, Zhu XJ (2018). BF211, a derivative of bufalin, enhances the cytocidal effects in multiple myeloma cells by inhibiting the IL-6/JAK2/STAT3 pathway. Int Immunopharmacol.

[12] Cheng CS, Wang J, Chen J, Kuo KT, Tang J, Gao H, Chen L, Chen Z, Meng Z (2019). New therapeutic aspects of steroidal cardiac glycosides: the anticancer properties of Huachansu and its main active constituent Bufalin. Cancer Cell Int.

[13] Woodford MR, Sager RA, Marris E, Dunn DM, Blanden AR, Murphy RL, Rensing N, Shapiro O, Panaretou B, Prodromou C (2017). Tumor suppressor Tsc1 is a new Hsp90 co-chaperone that facilitates folding of kinase and non-kinase clients. The EMBO journal.

[14] Isaacs JS, Xu W, Neckers L (2003). Heat shock protein 90 as a molecular target for cancer therapeutics. Cancer Cell.

[15] Li L, Chen NN, You QD, Xu XL (2021). An updated patent review of anticancer Hsp90 inhibitors (2013-present). Expert Opin Ther Pat.

[16] Weidenauer L, Wang T, Joshi S, Chiosis G, Quadroni MR (2017). Proteomic interrogation of HSP90 and insights for medical research. Expert Rev Proteomics.

[17] Hallett ST, Pastok MW, Morgan RML, Wittner A, Blundell K, Felletar I, Wedge SR, Prodromou C, Noble MEM, Pearl LH, Endicott JA (2017). Differential regulation of G1 CDK complexes by the Hsp90-Cdc37 chaperone system. Cell Rep.

[18] Nguyen MT (2016). Influence of post-translational modifications on Hsp90 activity and function.

[19] Blagg B, Mishra S, Khandelwal A, Bannerjee M, Balch M, Peng S, et al. Selective Inhibition of the Hsp90a isoform. Angew Chem Int Ed Engl. 2021;19:10547–51. 10.1002/anie.202015422 PMC8086817 33621416

[20] Mishra SJ, Liu W, Beebe K, Banerjee M, Kent CN, Munthali V, Koren J, Taylor JA, Neckers LM, Holzbeierlein J, Blagg BSJ (2021). The development of Hsp90β-selective inhibitors to overcome detriments associated with pan-Hsp90 inhibition. Journal of Medicinal Chemistry.

[21] Heider M, Eichner R, Stroh J, Morath V, Kuisl A, Zecha J, et al. The IMiD target CRBN determines HSP90 activity toward transmembrane proteins essential in multiple myeloma. Mol Cell. 2021;6:1170–86. 10.1016/j.molcel.2020.12.046 PMC7980223 33571422

[22] Zheng D, Liu W, Xie W, Huang G, Jiang Q, Yang Y, Huang J, Xing Z, Yuan M, Wei M (2021). AHA1 upregulates IDH1 and metabolic activity to promote growth and metastasis and predicts prognosis in osteosarcoma. Signal Transduction and Targeted Therapy.

[23] Shelton LB, Baker JD, Zheng D, Sullivan LE, Solanki PK, Webster JM, Sun Z, Sabbagh JJ, Nordhues BA, Koren J (2017). Hsp90 activator Aha1 drives production of pathological tau aggregates. Proceedings of the National Academy of Sciences.

[24] Zhan F, Huang Y, Colla S, Stewart JP, Hanamura I, Gupta S, Epstein J, Yaccoby S, Sawyer J, Burington B (2006). The molecular classification of multiple myeloma. Blood.

[25] Broyl A, Hose D, Lokhorst H, de Knegt Y, Peeters J, Jauch A, Bertsch U, Buijs A, Stevens-Kroef M, Beverloo HB (2010). Gene expression profiling for molecular classification of multiple myeloma in newly diagnosed patients. Blood.

[26] Gu C, Lu T, Wang W, Shao M, Wei R, Guo M, et al. RFWD2 induces cellular proliferation and selective proteasome inhibitor resistance by mediating P27 ubiquitination in multiple myeloma. Leukemia. 2020;6:1803–7. 10.1038/s41375-020-01033-z 32901100

[27] Goldschmidt H, Lokhorst HM, Mai EK, van der Holt B, Blau IW, Zweegman S, Weisel KC, Vellenga E, Pfreundschuh M, Kersten MJ (2018). Bortezomib before and after high-dose therapy in myeloma: long-term results from the phase III HOVON-65/GMMG-HD4 trial. Leukemia.

[28] Gu C, Lu T, Wang W, Shao M, Wei R, Guo M, Li R, Qiao L, Hu Y, Zhan F (2021). RFWD2 induces cellular proliferation and selective proteasome inhibitor resistance by mediating P27 ubiquitination in multiple myeloma. Leukemia.

[29] Yang Y, Guan D, Lei L, Lu J, Liu JQ, Yang G, Yan C, Zhai R, Tian J, Bi Y (2018). H6, a novel hederagenin derivative, reverses multidrug resistance in vitro and in vivo. Toxicol Appl Pharmacol.

[30] Verba K, Wang R, Arakawa A, Liu Y, Shirouzu M, Yokoyama S, Agard D (2016). Atomic structure of Hsp90-Cdc37-Cdk4 reveals that Hsp90 traps and stabilizes an unfolded kinase. Science.

[31] Meyer P, Prodromou C, Liao C, Hu B, Roe SM, Vaughan CK, Vlasic I, Panaretou B, Piper PW, Pearl LH (2004). Structural basis for recruitment of the ATPase activator Aha1 to the Hsp90 chaperone machinery. EMBO J.

[32] UniProt Consortium T (2018). UniProt: the universal protein knowledgebase. Nucleic Acids Res.

[33] Summa CM, Levitt M (2007). Near-native structure refinement using in vacuo energy minimization. Proc Natl Acad Sci U S A.

[34] Halgren TA (2009). Identifying and characterizing binding sites and assessing druggability. J Chem Inf Model.

[35] Halgren T (2007). New method for fast and accurate binding-site identification and analysis. Chem Biol Drug Des.

[36] Friesner RA, Banks JL, Murphy RB, Halgren TA, Klicic JJ, Mainz DT, Repasky MP, Knoll EH, Shelley M, Perry JK (2004). Glide: a new approach for rapid, accurate docking and scoring. 1. Method and assessment of docking accuracy. J Med Chem.

[37] Gu C, Yang Y, Sompallae R, Xu H, Tompkins VS, Holman C, et al. FOXM1 is a therapeutic target for high-risk multiple myeloma. Leukemia. 2015;4:873–82. 10.1038/leu.2015.334 PMC4826574 26648534

[38] Gu C, Holman C, Sompallae R, Jing X, Tomasson M, Hose D, Seckinger A, Zhan F, Tricot G, Goldschmidt H (2018). Upregulation of FOXM1 in a subset of relapsed myeloma results in poor outcome. Blood Cancer J.

[39] Gu C, Cheng H, Yang H, Bian Y, Wang Y, Zhang Y, et al. MK2 is a therapeutic target for high-risk multiple myeloma. Haematologica. 2018;6:1774–77. 10.3324/haematol.2017.182121 PMC8168486 29567777

[40] Su S, Yang Z, Gao H, Yang H, Zhu S, An Z, Wang J, Li Q, Chandarlapaty S, Deng H (2019). Potent and preferential degradation of CDK6 via proteolysis targeting chimera degraders. Journal of Medicinal Chemistry.

[41] Yuan K, Wang X, Dong H, Min W, Hao H, Yang P (2021). Selective inhibition of CDK4/6: a safe and effective strategy for developing anticancer drugs. Acta Pharmaceutica Sinica B.

[42] Shaughnessy JD, Qu P, Usmani S, Heuck CJ, Zhang Q, Zhou Y, Tian E, Hanamura I, van Rhee F, Anaissie E (2011). Pharmacogenomics of bortezomib test-dosing identifies hyperexpression of proteasome genes, especially PSMD4, as novel high-risk feature in myeloma treated with total therapy 3. Blood.

[43] Xu H, Han H, Song S, Yi N, Qian C, Qiu Y, Zhou W, Hong Y, Zhuang W, Li Z (2019). Exosome-transmitted PSMA3 and PSMA3-AS1 promote proteasome inhibitor resistance in multiple myeloma. Clin Cancer Res.

[44] Weiss A, Neubauer MC, Yerabolu D, Kojonazarov B, Schlueter BC, Neubert L, Jonigk D, Baal N, Ruppert C, Dorfmuller P (2019). Targeting cyclin-dependent kinases for the treatment of pulmonary arterial hypertension. Nature Communications.

[45] Verba KA, Wang RY, Arakawa A, Liu Y, Shirouzu M, Yokoyama S, Agard DA (2016). Atomic structure of Hsp90-Cdc37-Cdk4 reveals that Hsp90 traps and stabilizes an unfolded kinase. Science.

[46] Krukenberg KA, Street TO, Lavery LA, Agard DA (2011). Conformational dynamics of the molecular chaperone Hsp90. Q Rev Biophys.

[47] Serwetnyk M, Blagg B (2021). The disruption of protein-protein interactions with co-chaperones and client substrates as a strategy towards Hsp90 inhibition. Acta Pharmaceutica Sinica B.

[48] Botelho AFM, Pierezan F, Soto-Blanco B, Melo MM (2019). A review of cardiac glycosides: structure, toxicokinetics, clinical signs, diagnosis and antineoplastic potential. Toxicon.

[49] Guo M, Sun D, Fan Z, Yuan Y, Shao M, Hou J, Zhu Y, Wei R, Zhu Y, Qian J (2019). Targeting MK2 is a novel approach to interfere in multiple myeloma. Front Oncol.

[50] Zhou W, Yang Y, Xia J, Wang H, Salama ME, Xiong W, Xu H, Shetty S, Chen T, Zeng Z (2013). NEK2 induces drug resistance mainly through activation of efflux drug pumps and is associated with poor prognosis in myeloma and other cancers. Cancer Cell.

[51] Yang Y, Shi J, Tolomelli G, Xu H, Xia J, Wang H, Zhou W, Zhou Y, Das S, Gu Z (2013). RARalpha2 expression confers myeloma stem cell features. Blood.

[52] Sun D, Tao W, Zhang F, Shen W, Tan J, Li L, Meng Q, Chen Y, Yang Y, Cheng H (2020). Trifolirhizin induces autophagy-dependent apoptosis in colon cancer via AMPK/mTOR signaling. Signal Transduction and Targeted Therapy.

[53] Oravecz M, Mészáros J. [Traditional Chinese medicine: theoretical background and its use in China]. Orv Hetil 2012; 153:723-731. 10.1556/OH.2012.29365 22564283

[54] Dutta Gupta S, Pan CH (2020). Recent update on discovery and development of Hsp90 inhibitors as senolytic agents. Int J Biol Macromol.

