# Correction: AHSA1 is a promising therapeutic target for cellular proliferation and proteasome inhibitor resistance in multiple myeloma

**DOI:** 10.1186/s13046-023-02672-7

**Published:** 2023-04-19

**Authors:** Chunyan Gu, Yajun Wang, Lulin Zhang, Li Qiao, Shanliang Sun, Miaomiao Shao, Xiaozhu Tang, Pinggang Ding, Chao Tang, Yuhao Cao, Yanyan Zhou, Mengjie Guo, Rongfang Wei, Nianguang Li, Yibei Xiao, Jinao Duan, Ye Yang

**Affiliations:** 1 Nanjing Hospital of Chinese Medicine affiliated to Nanjing University of Chinese Medicine, Nanjing, 210023 China; 2 School of Medicine & Holistic Integrative Medicine, Nanjing University of Chinese Medicine, Nanjing, 210023 China; 3 School of Pharmacy, Nanjing University of Chinese Medicine, Nanjing, 210023 China; 4 School of Pharmacy, China Pharmaceutical University, Nanjing, 211198 China; 5 State Administration of Traditional Chinese Medicine Key Laboratory of Chinese Medicinal Resources Recycling Utilization, Jiangsu Collaborative Innovation Center of Chinese Medicinal Resources Industrialization, Nanjing University of Chinese Medicine, Nanjing, 210023 China

**Correction** **:** **J Exp Clin Cancer Res 41, 11 (2022)**

**https://doi.org/10.1186/s13046-021-02220-1**

Following publication of the original article [1], an error was identified Figs. 1, 2, and 4 specifically:

- Fig. 1e - The numbers of the primary samples were mistyped, which should be consistent with Fig. 2B
- Fig. 2d - The number of the recurrent samples was mistyped, which should be 6 instead of 15
- Fig. 4f - Misplaced images and one incorrect image were used

Furthermore, Figure 1 caption has to be updated. The correct figures and the correct Figure 1 caption are presented below:

**Fig. 1:**
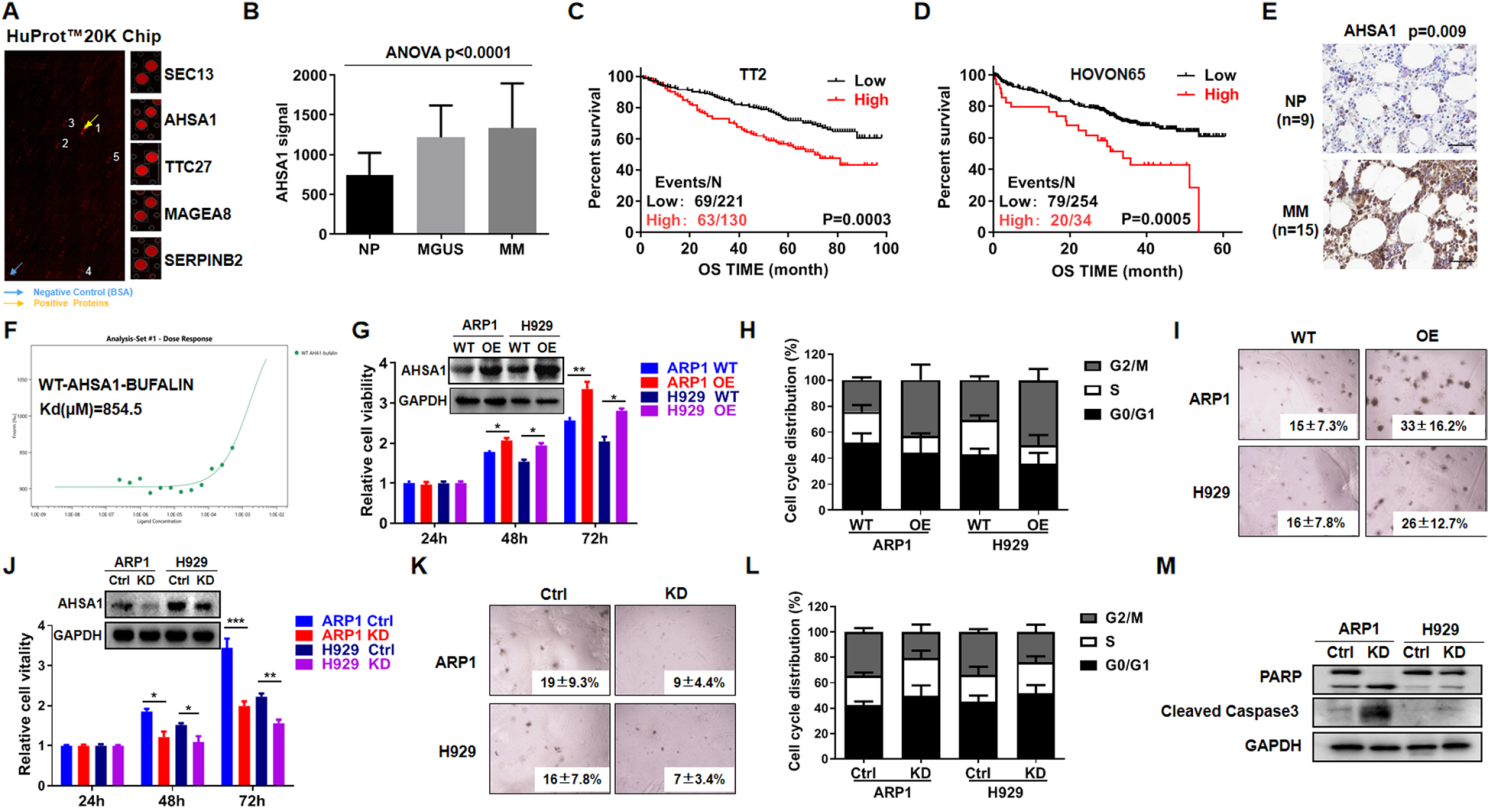
Elevated AHSA1 expression confers poor survival of MM patients and promotes MM cell proliferation. (**A**) HuProt™20 K Proteome Microarray Chip indicated the top 5 protein targets binding to Bufalin. The yellow arrow indicated positive protein interacted with Bufalin, and the blue arrow represented the negative control. (**B**) Among the top 5 proteins, AHSA1 was the exclusive gene. The signal level of AHSA1 was shown on the y-axis. Patients designated as healthy donors with normal bone marrow plasma cells (NP, n = 22), monoclonal gammopathy of undetermined significance (MGUS, n = 44) or multiple myeloma (MM, n = 351) were sorted on the x-axis. (**C-D**) Increased AHSA1 mRNA expression was positively associated with poor overall survival (OS) in first diagnosis and relapsed MM patients from (C) TT2 and (D) HOVON65 patient cohort. Events/N means events of death/total patients. (**E**) Representative Immunohistochemistry staining on primary MM samples (n = 15) and normal controls (n = 9). (**F**) Microscale thermophoresis (MST) analysis for the interaction of Bufalin with human AHSA1 recombination protein. (**G**) Validation of AHSA1 overexpression in AHSA1*-*OE ARP1 and H929 cells relative to control cells. (**H**) Cell cycle analysis for WT and AHSA1-OE cells. (**I**) Representative images of cell colonies of WT and AHSA1-OE cells in soft agar. (**J**) Confirmation of AHSA1 protein knockdown in ARP1 and H929 cells after transfection with AHSA1 shRNA. (**K**) Representative images of cell colonies of WT and AHSA1-KD cells in soft agar. (**L**) Cell cycle analysis for WT and AHSA1-KD cells. (**M**) WB analysis of PARP and Caspase 3. The data are expressed as mean ± SD.*p < 0.05, **p < 0.01, ***p < 0.001

**Fig. 2:**
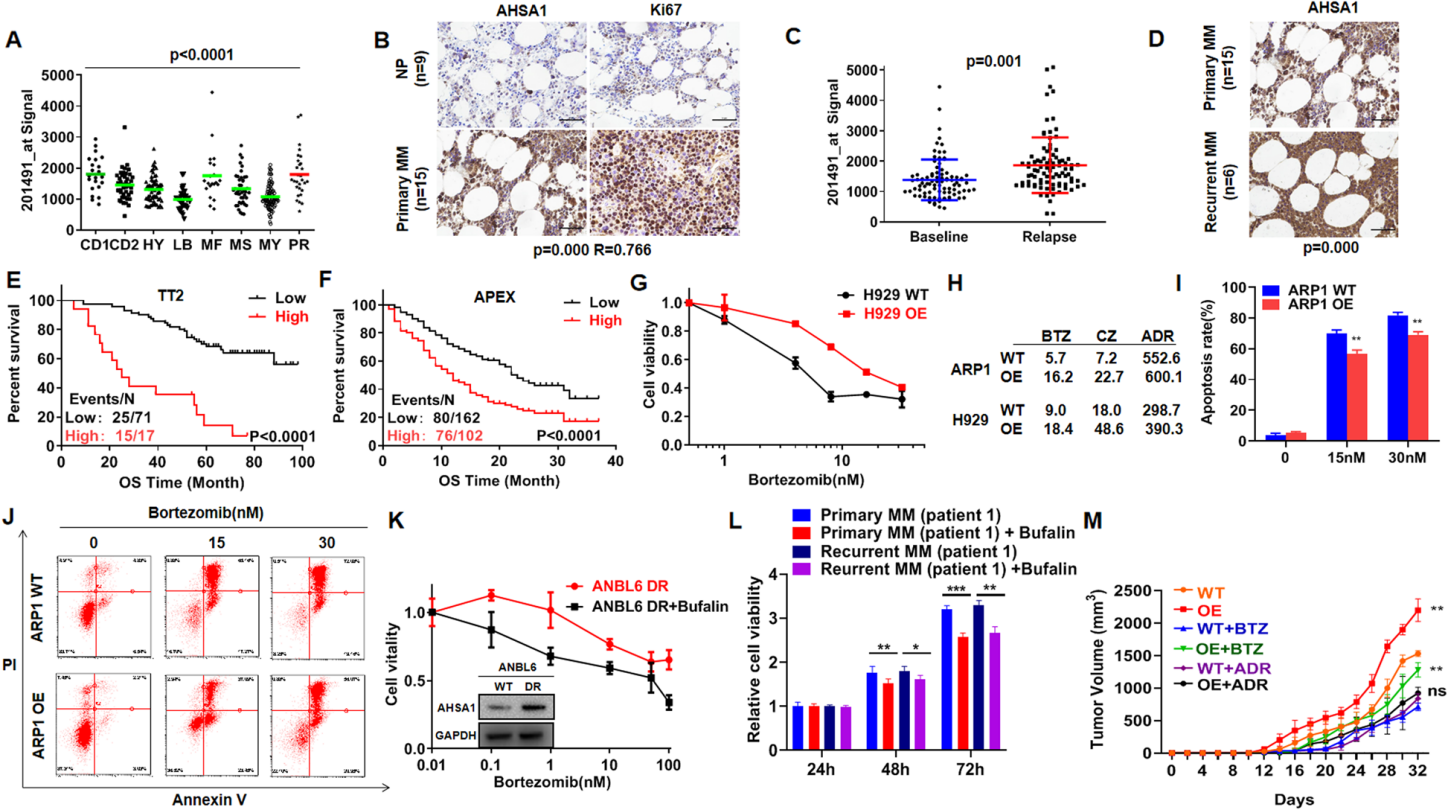
AHSA1 is a high-risk MM marker and induces proteasome inhibitor resistance in vitro and in vivo. **A** Box plot representing AHSA1 expression in eight MM risk subgroups from TT2 patient cohort. **B** IHC staining of AHSA1 and Ki67 expressions in MM patient samples. **C** AHSA1 mRNA expression in paired patient MM samples collected at first diagnosis and relapse stage. **D** IHC staining of AHSA1 expression in the relapsed samples and the corresponding samples from first diagnosis. **E–F** Elevated AHSA1 expression was correlated with decreased OS in relapsed patients from the (**E**) TT2 and (**F**) APEX cohorts by long-term following up. **G** Effects of Bortezomib on cell viability of H929 cells with or without overexpression of AHSA1. **H** IC50 values of BTZ, CZ and ADR in MM cells with or without overexpression of AHSA1. **I** The rate of BTZ-induced apoptosis was shown in the histogram. **J** Effects of BTZ on cell apoptosis in ARP1 cells with or without overexpression of AHSA1. **K** Effects of Bufalin on cell viability in ANBL6 DR (Bortezomib-resistant) cells. **L** Effects of Bufalin (60 nM) on the cell viability of flow MRD-positive peripheral cells from first diagnosed and relapsed MM patients. **M** Time course of tumor growth in ARP1 AHSA1 WT/OE xenografts taken from NOD-SCID mice treated with vehicle, BTZ, or ADR. The data are expressed as mean $\pm$ SD.*p < 0.05, **p < 0.01, ***p < 0.001

**Fig. 4:**
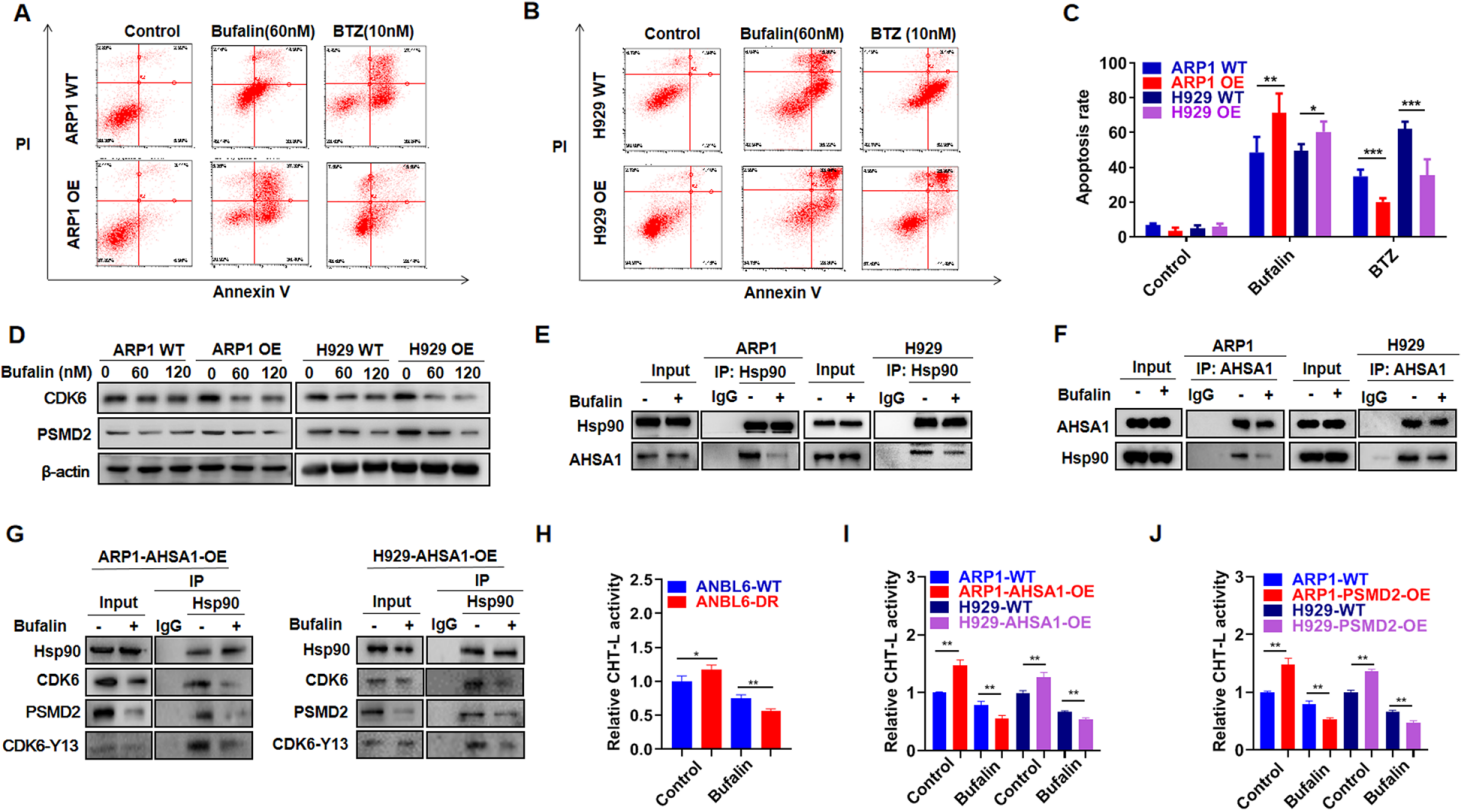
Bufalin decreases cellular proliferation and PI resistance induced by AHSA1/HSP90 in MM cells. **A-B** Effects of Bufalin (60 nM) and BTZ (10 nM) incubation for 48 h on cell apoptosis of ARP1 (**A**) and H929 (**B**) WT and AHSA1-OE cells. **C** The rate of drug-induced apoptosis was shown in the histogram. **D** Effects of Bufalin on the expression of CDK6 and PSMD2 in ARP1 and H929 WT and AHSA1-OE cells. **E–F** Co-IP assay revealed that Bufalin interfered the interaction between HSP90 and AHSA1 in ARP1 and H929 cells. **G** Co-IP assay confirmed the interaction between AHSA1, HSP90, CDK6, PSMD2 and the activated form of CDK6, phosphorylation of Y13 site at CDK6. **H-J** Proteasome activity assay verified that Bufalin inhibited proteasome activity in (**H**) ANBL6 WT/DR cells, **I** ARP1 and H929 AHSA1 WT/OE cells and **J** PSMD2 WT/OE cells. The data are expressed as mean $\pm$ SD.*p < 0.05, **p < 0.01, ***p < 0.001

The correction do not affect the overall Conclusion of the article.
