# Supplementary figures and images for: AHSA1 is a promising therapeutic target for cellular proliferation and proteasome inhibitor resistance in multiple myeloma
Source: J Exp Clin Cancer Res. 2022 Jan 6;41:11. doi: 10.1186/s13046-021-02220-1 (PMC8734095; doi:10.1186/s13046-021-02220-1)

Supplementary Figure 1

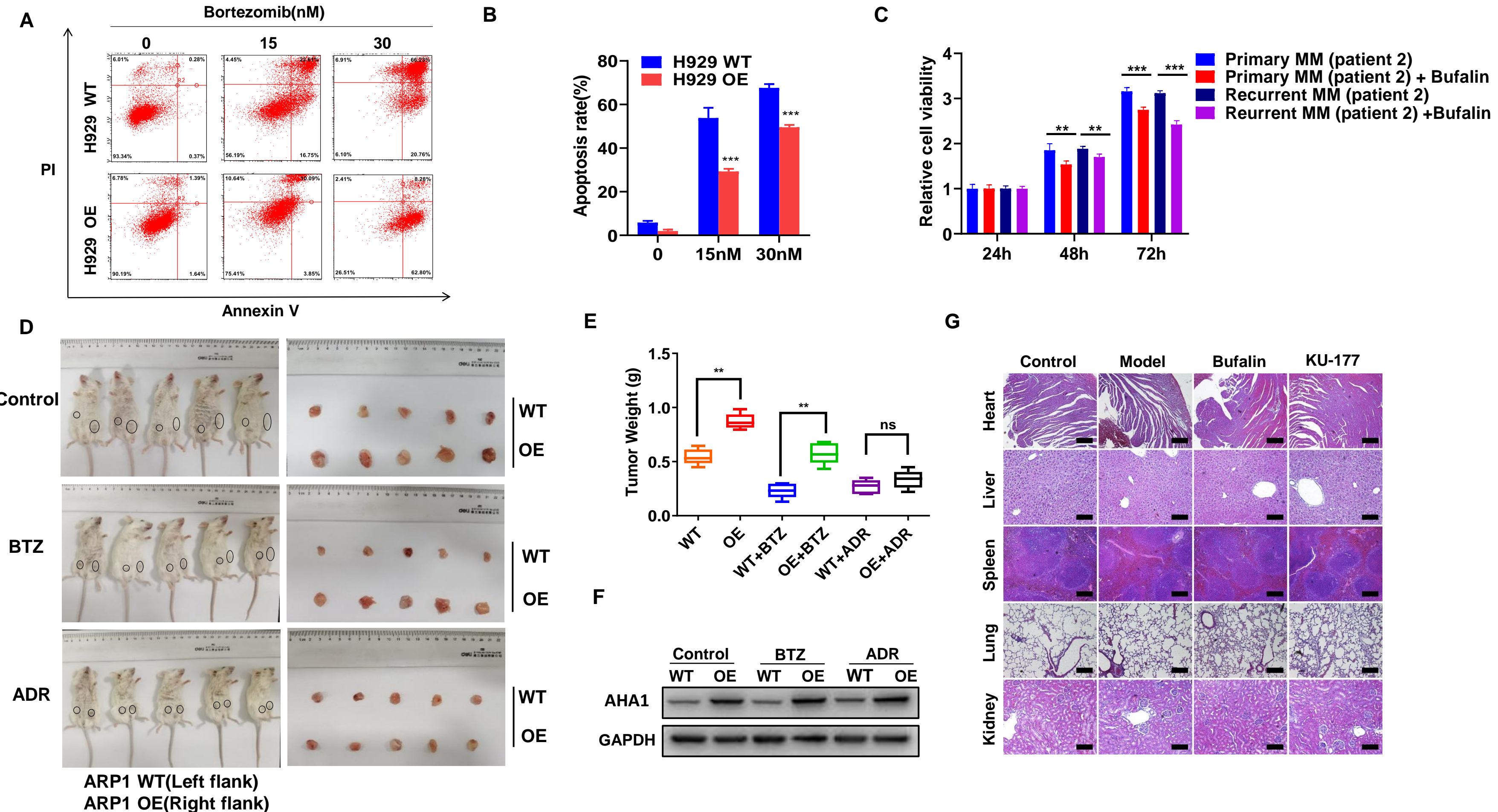

Supplement: Supplementary file 1 — Additional file 1: Figure S1. AHSA1 induces proteasome inhibitor resistance in vitro and in vivo. (A) Effects of Bortezomib on cell apoptosis in H929 cells with or without overexpression of AHSA1. (B) The analysis of bortezomib-induced apoptosis. (C) Effects of Bufalin (60nM) on the cell viability in flow MRD-positive peripheral cells from first diagnosed and relapsed MM patients. (D) Photographic images of ARP1 AHSA1 WT/OE xenografts taken from NOD-SCID mice treated with vehicle, BTZ, or ADR. (E) Mean tumor weight in the six experimental groups at day 32 after implantation of MM cells. (F) Western blot analysis of AHSA1 expression of the tumors in the experimental groups. (G) Images of representative HE staining of heart, liver, spleen, lung and kidney from control and 5TMM mouse model with or without Bufalin or KU-177 treatment. [file 13046_2021_2220_MOESM1_ESM.pdf]
